# Retinal structural-vascular-functional relationship using optical coherence tomography and optical coherence tomography – angiography in myopia

**DOI:** 10.1186/s40662-019-0133-6

**Published:** 2019-03-07

**Authors:** Ramesh Venkatesh, Shivani Sinha, Deepika Gangadharaiah, Santosh G. K. Gadde, Ashwin Mohan, Rohit Shetty, Naresh Kumar Yadav

**Affiliations:** 1 Department of Retina and Vitreous, Narayana Nethralaya, #121/C, 1st R Block, Chord RoadRajaji Nagar, Bengaluru, 560010 India; 2 Department of Cornea and Refractive surgery, Narayana Nethralaya, #121/C, 1st R Block, Chord Road, Rajaji Nagar, Bengaluru, 560010 India

**Keywords:** Myopia, Axial elongation, Spherical equivalent, OCT, Optical coherence tomography angiography, Structure, Visual acuity

## Abstract

**Background:**

To examine the retinal structure–vascular-function relationship using optical coherence tomography (OCT) and optical coherence tomography angiography (OCTA) in myopia.

**Methods:**

This was a prospective cross-sectional study comprising 86 eyes of 45 individuals with varying axial lengths and spherical equivalents and no posterior segment abnormalities. All eyes underwent optical coherence tomography with the Spectralis SD-OCT and OCTA with RTVue-XR Avanti; Optovue. Individual macular retinal layer thicknesses and flow areas and vessel densities were measured on OCT and OCTA, respectively. Linear correlations were made between the macular layer thicknesses, flow areas and vessel densities with axial length, spherical equivalent and visual acuity.

**Results:**

The participants’ mean ages were 33.34 ± 14.45 years, mean spherical equivalent refractions were − 7.17 ± 5.71 D and axial lengths were 25.95 ± 2.41 mm. There were significant positive correlations of foveal angle (r = 0.757, *p* = 0.001), inner retinal (r = 0.764, p = 0.001) and outer plexiform layer (r = 0.771, p = 0.001) thickness on OCT and vessel densities in deep capillary plexus (r = 0.313, *p* = 0.003) on OCTA with axial length and negative correlations with spherical equivalents and visual acuity. Significant negative correlations of outer nuclear layer (r = − 0.560, *p* = 0.03) and photoreceptor outer segment layer thickness (r = − 0.856, *p* < 0.001) were noted on OCT with axial length and positive correlations with spherical equivalents and visual acuity.

**Conclusion:**

The lateral retinal stretching in myopia could possibly explain the correlation between retinal layer thickness, vascular density and visual acuity in these eyes. Further research is required to investigate this.

## Background

Myopia is one of the common refractive errors worldwide [1]. High myopia is characterised by abnormal axial elongation and scleral thinning [2]. With increase in axial length, the retina shows microstructural degenerative changes, especially at the posterior pole. Myopia is a risk factor for several retinal pathologies such as retinal detachment, macular holes, choroidal neovascularization, and retinoschisis [3, 4]. Thus, complications related to myopia are one of the leading causes of visual impairment. As a result, early detection of the changes in intraretinal structures of a myopic eye is of utmost importance. Identifying the abnormal patterns of retinal structures will help in assessing early-stage, myopia-related complications. High-resolution optical coherence tomography (OCT) has been useful in imaging and measurement of retinal thickness in vivo, and to evaluate structural change associated with retinal diseases [5, 6]. Recently, with improvements in axial resolution and image processing methods, OCT-based in vivo macular thickness measurements of the intraretinal layers have been made possible. Automated layer segmentation algorithms have been developed to analyse the individual intraretinal layer thicknesses [7–10]. These studies have shown that the thickness of specific retinal layers can help to diagnose and monitor pathologic changes in the macula resulting from retinal diseases [11], glaucoma [12] and optic neuropathy [13]. Several studies have used OCT to investigate relationships between variations in macular thickness and myopia [14–17]. The inner retinal layer thicknesses, namely of the retinal nerve fibre layer (NFL), ganglion cell layer (GCL), and inner plexiform layer (IPL) were reported by some authors to be thinner in myopic eyes compared to normal eyes [18]. Furthermore, the thickness of the outer retinal layers, including the outer plexiform (OPL), outer nuclear (ONL) and photoreceptor layers, vary according to the axial length (AL) [19]. However, the results of the above studies are controversial because the characteristic of macular intraretinal layer thickness in myopia remains unclear. Additionally, it is important to determine if there is any correlation between the retinal structure and visual acuity in myopic patients.

In myopia-related retinal disorders, in addition to the retinal microstructural changes, the retinal microvasculature also contributes to the visual function. Hence, much attention has been paid to the changes in retinal microvasculature because it serves as a direct source of oxygen and nutrients for the neuro retinal layers. Earlier studies have found that high myopia is frequently associated with retinal vascular alterations, such as decreased retinal vessel density or increased vessel resistance, which can be detected by colour doppler imaging or fundus photography [20–23]. Optical coherence tomography angiography (OCTA) is a novel non-invasive technology that provides depth-resolved visualization of the retinal and choroidal microvasculature without the need for dye injection by using phase or amplitude decorrelation to identify the motion contrast of blood flow [24, 25]. Previous studies have shown great intra- and inter-visit repeatability and reproducibility of OCTA in the optic nerve head and macular microvascular perfusion measurements [26, 27]. However, contrasting results have been reported in studies using this technology. Wang et al.[28] evaluated the parapapillary and parafoveal microvascular perfusion using OCTA and found a decreased vessel density in the parapapillary area, but not in the parafoveal area, of eyes with high myopia in comparison with emmetropic eyes. Mo et al.[29] reported similar results. Moreover, they observed a decreased macular flow density in pathological myopia compared with high myopia and emmetropia. Eyes with myopia and glaucoma showed a progressive decrease in the peripapillary perfused capillary density on OCTA compared to eyes with either myopia or glaucoma [30]. On the contrary, Yang et al.[31] and Li et al.[32] showed a decreased parafoveal microvascular density in eyes with high myopia when compared to those with mild myopia and emmetropia. As far as the authors are aware, there have been limited studies on structure-vasculature-function relationship in myopic eyes [33–36]. The hypothesis for the current study is that intra retinal structural changes on OCT and retinal microvascular changes on OCTA are responsible for the visual function with higher grades of myopia. The purpose of the current study was to analyse the variations in the individual macular retinal layer thicknesses using the automated layer segmentation algorithm of the high-resolution OCT and retinal microvasculature using the OCTA and study its relationship with axial length, visual acuity and spherical refraction.

## Methods

After obtaining the approval from the institutional review board and ethics committee, a total of 86 eyes of 45 Indian patients were recruited between October 2017 to March 2018 in this prospective cross-sectional study. The study was conducted in accordance with the tenets of the declaration of Helsinki. A written informed consent was obtained from each participant. The inclusion criteria were as follows: age ≥ 18 years, astigmatism within ±2.00 D, intraocular pressure (IOP) less than 21 mmHg, normal anterior chamber angles, and no optic disk abnormalities. Participants with findings of myopic maculopathy like macular hole, epiretinal membrane and foveoschisis were excluded from the study. Participants with history of ocular trauma or intraocular surgery, and any ocular or systemic disorders (such as glaucoma or diabetes mellitus) which might affect the ocular circulation were excluded. Participants where either the OCT or OCTA was not possible to procure were also excluded from the study.

All subjects were required to provide a detailed medical history and undergo a thorough ophthalmic examination including measurement of refractive status, Snellen’s best corrected visual acuity (VA), IOP measurement using Goldman applanation tonometry, slit-lamp examination, axial length (AL) measurement using optical low-coherence reflectometry (Lenstar 900; Haag-Streit Diagnostics, Koeniz, Switzerland). Individual macular retinal layer thicknesses were measured using OCT (Heidelberg Spectralis, Germany). Retinal microvascular findings were noted using the OCTA (Avanti, Optovue).

### Retinal imaging using OCT

The macular total thickness and individual retinal layer thicknesses were measured with spectral-domain OCT (Spectralis, Heidelberg Engineering, Heidelberg, Germany). Macular volumetric assessments consisting of horizontal axial scans with 512 A-scans per line with scanning area 6 × 6 mm, 25 scan patterns centred at the fovea, were performed. The automatic real-time function was employed and nine images at the same location were captured and averaged automatically by the instrument software to decrease image noise-to-signal ratio and improve the image quality. The scan with higher signal and image quality was selected for further analysis.

### Measurements using OCT image

**Fig. 1:**
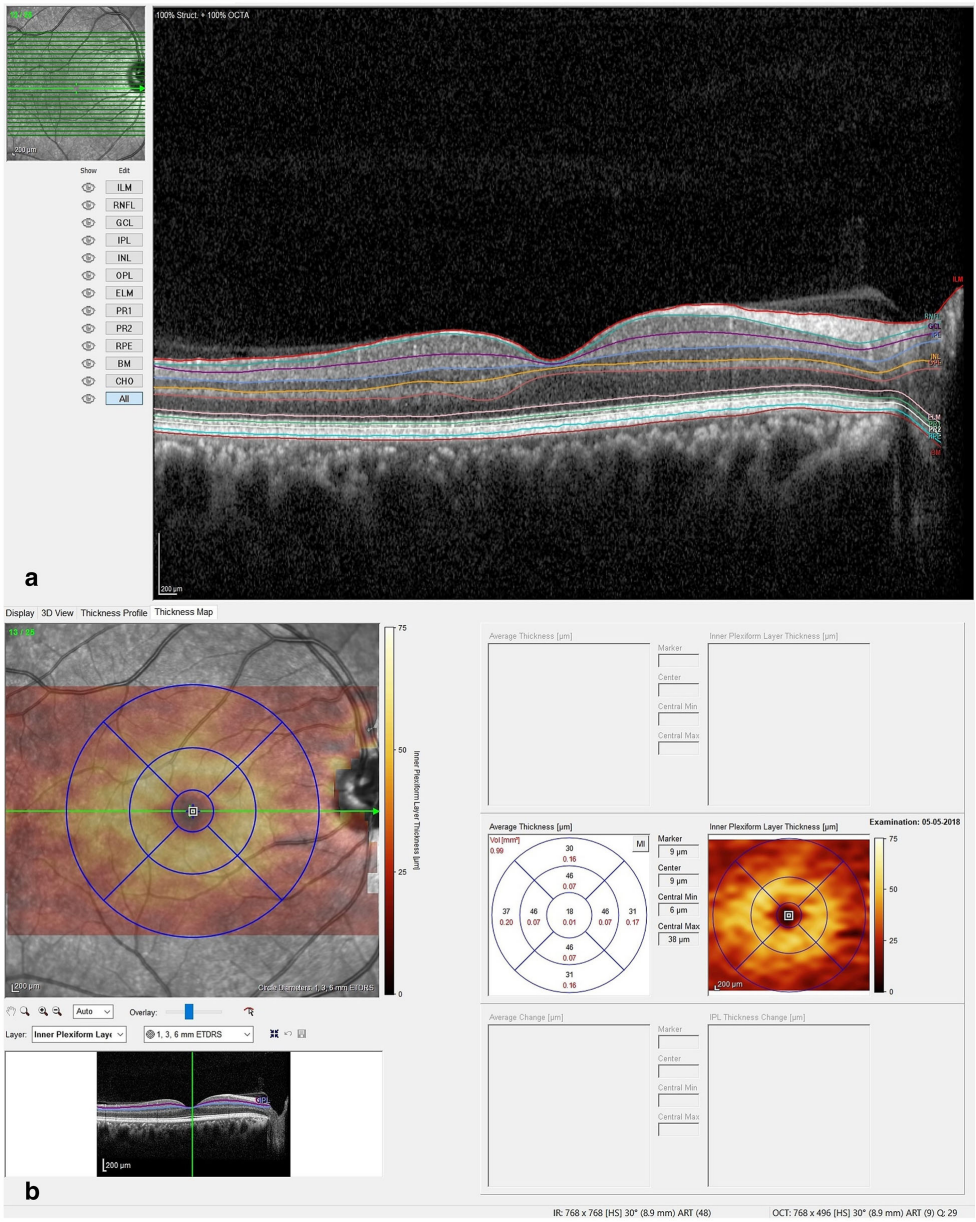
Retinal layer segmentation on Heidelberg Spectralis SD-OCT machine. **a** Automated retinal layer segmentation using the Heidelberg Spectralis machine. **b** Measurement of individual retinal layers using the 1 mm, 3 mm and 6 mm ETDRS grid

All thickness measurements were made on the SD-OCT using the automated layer segmentation software (Fig. 1a). In cases of automatic layer misalignment, manual alignment was possible by SD-OCT software before automatic measurements. A semiautomated approach was incorporated into the algorithm to correct for any minor segmentation errors. In addition, all the boundaries were checked by visual inspection performed by 2 of the authors (RV and SSH). For evaluation of the macular area, each macular thickness map was divided into nine regions suggested by the Early Treatment for Diabetic Retinopathy Study [37] including a 1 mm diameter central disc and an inner and outer ring, each divided into four quadrants, with diameters of 3 and 6 mm, respectively. OCT delineates every macular layer, and we measured the thickness of individual retinal layers (Fig. 1b). Neuro-sensory retina was segmented into 7 intraretinal layers, namely: 1) retinal nerve fibre layer (NFL) 2) ganglion cell layer (GCL) 3) inner plexiform layer (IPL) 4) inner nuclear layer (INL) 5) outer plexiform layer (OPL) 6) outer nuclear layer (ONL) and 7) outer segment of photoreceptors (OS). Average macular layer thicknesses were calculated by averaging the inner and outer segments, excluding the foveal region in each quadrant (superior, inferior, temporal, nasal). For the ease of understanding, we combined the superficial retinal layers, namely NFL, GCL, IPL and INL into one group as inner retinal layer (IRL).

### Measurement of foveal angle

**Fig. 2:**
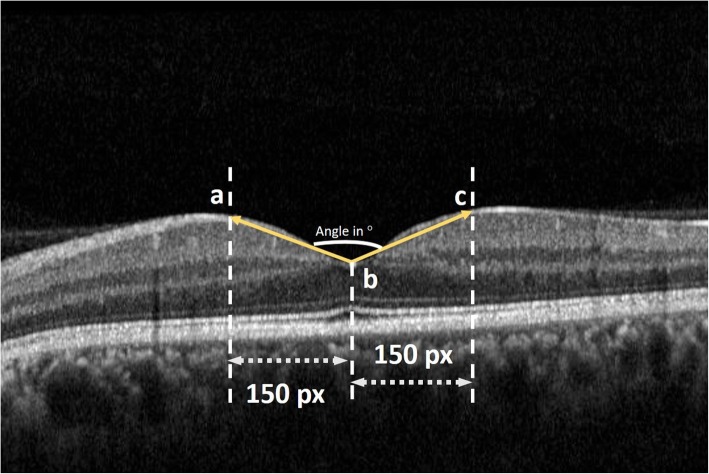
Image demonstrating the technique for foveal angle measurement using Image J

The same OCT scan image was saved in the .jpg/.jpeg format and then exported to image J (http://imagej.nih.gov/ij/; provided in the public domain by the National Institutes of Health, Bethesda, MD, USA - version 1.51) to measure the foveal angle. The procedure of measuring foveal angle in schematically described in Fig. 2.

### Retinal microvasculature imaging with OCTA

**Fig. 3:**
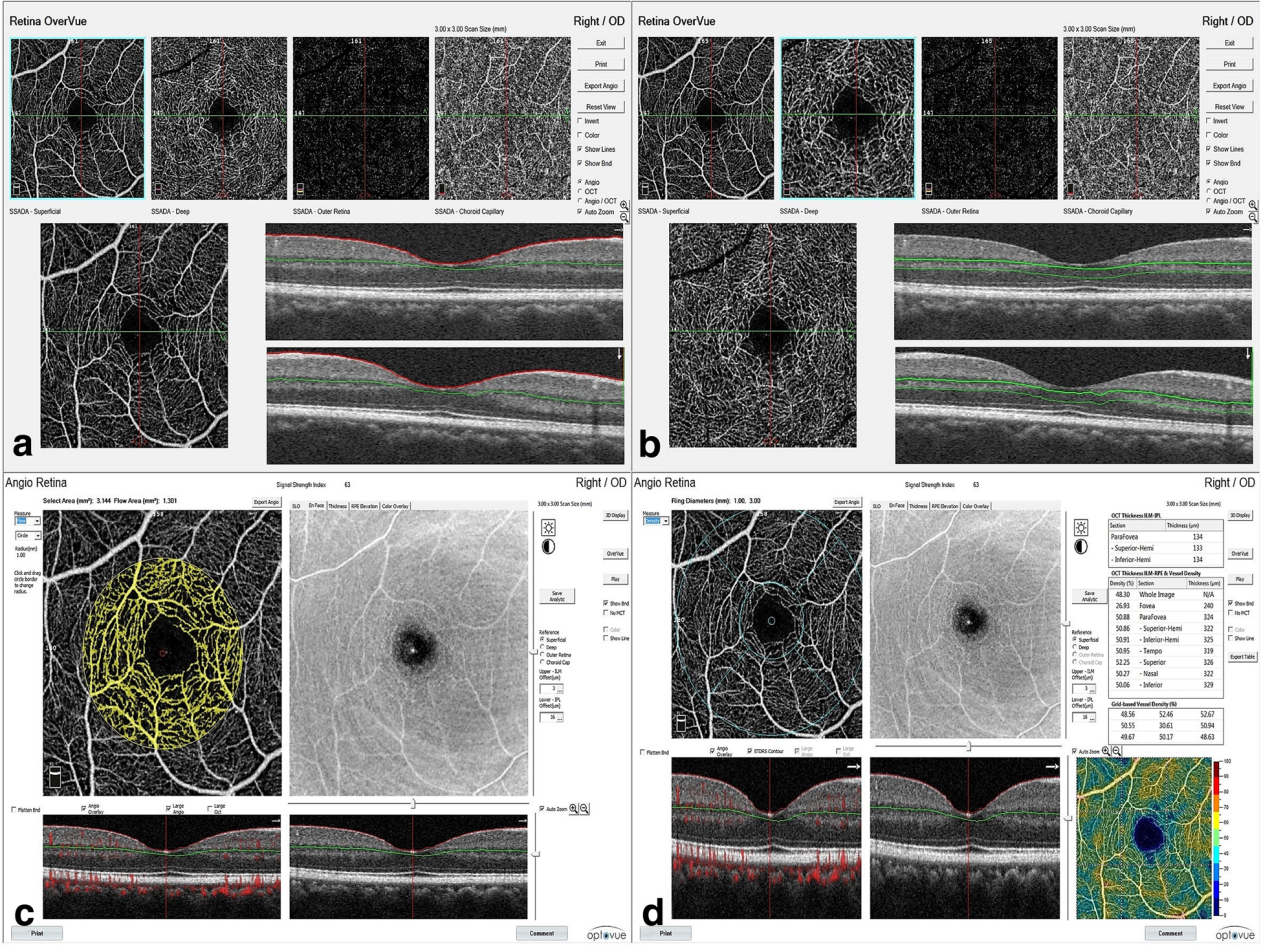
Segmentation technique on OCT-angiography (OCTA) and measurements of flow area index and vessel density using the AngioVue software on OCTA. **a** and **b** Automated segmentation of the superficial and deep capillary plexuses using the Avanti spectral domain OCT (RTVue-XR Avanti; Optovue, Fremont, CA, USA). **c** and **d** Images depicting the measurements of flow area index and vessel density on OCTA

With a built-in AngioVue software, the Avanti spectral domain OCT (RTVue-XR Avanti; Optovue, Fremont, CA, USA) was used for retinal vessel imaging. Specifically, the signal for kinetic retinal blood was obtained using the SSADA algorithm, an amplitude-based OCT angiography method, which provided decorrelation values for each of the vessel so that we could quantitatively evaluate the retinal vasculature [25]. In this context, microvessel density and flow index in the superficial and deep capillary plexus in the parafoveal region were calculated. Vessel density is defined as percentage area occupied by vessels in the segmented area. Flow index is defined as the average flow signal (which is correlated with flow velocity) in a selected area. The parafoveal region was defined as a 1.9 mm wide annulus surrounding the fovea with an inner diameter of 0.6 mm and an outer diameter of 2.5 mm. The entire enface microvasculature was evaluated in the 3 × 3 mm area of the parafoveal region. The retina was automatically separated into various layers by the AngioVue software. It should be noted that we used the measurements of superficial and deep vascular layers for further analysis. The superficial capillary plexus (SCP) extended from 3 μm below the internal limiting membrane (ILM) to 15 μm below the IPL. The deep capillary plexus (DCP) extended from 16 μm below the IPL to 69 μm below the IPL. Superficial retinal microvascular density was calculated separately in four sectors (superior, inferior, temporal, and nasal) in the parafoveal area based on the early treatment diabetic retinopathy study (ETDRS) contour. The average density of the parafoveal area was measured. The flow index in the parafoveal region in the SCP was measured. Similarly, the flow index and vessel density were calculated in the deep capillary layer plexus as well. All OCTA scans were performed by one proficient examiner who was unaware of the other ocular data of the participants (Fig. 3). All OCTA scans with signal strength index ≥60, proper segmentation, and with no artefacts were evaluated by one author (RV).

### Statistical analysis

Normal distribution of quantitative variables was checked using the D’Agostino & Pearson omnibus normality test. Refraction data were converted to spherical equivalent (SE), which were calculated as the spherical dioptric power plus one-half of the cylindrical dioptric power. Snellen’s VA was converted to approxETDRS letters using the formula 85 + 50 × log(Snellen fraction) [38]. Correlations between the foveal angle and age were analysed. Associations between macular thicknesses, vessel densities and flow indices in the superficial and deep capillary plexuses and AL and SE were determined with Pearson’s correlation tests. A correlation (r) value of 0 means no correlation between the 2 variables while values closer to − 1 indicate strong negative correlation and values closer to + 1 indicate strong positive correlation. We used multivariable linear regression analysis to assess the relationship between various macular retinal layer thickness, vessel densities and flow indices as independent variables and AL, SE and VA as dependent variables. All data were analysed with GraphPad Prism software (version 7.05). *P* values <.05 were considered statistically significant.

## Results

Eighty-six eyes from 45 healthy subjects with SEs ranging from 1.75 D to − 20 D (mean: − 7.17 ± 5.71 D) and ALs ranging from 21.77 mm to 32.28 mm (mean: 25.95 ± 2.41 mm) were included in the analysis (Table 1). Minor automated segmentation misalignment was noted in 6 eyes and was corrected manually before the measurements were recorded.

**Table 1:** Baseline Data

Number of eyes, (subjects)	86 (45)
Age, years (range)	33.34 ± 14.45 (10–44)
Sex, Male/Female	19/26
Axial length, mm (range)	25.95 ± 2.41 (21.77–32.28)
Spherical equivalent, D (range)	−7.17 ± 5.71 (1.75–20)

### Relationship between thickness profile and AL, SE and VA

**Fig. 4:**
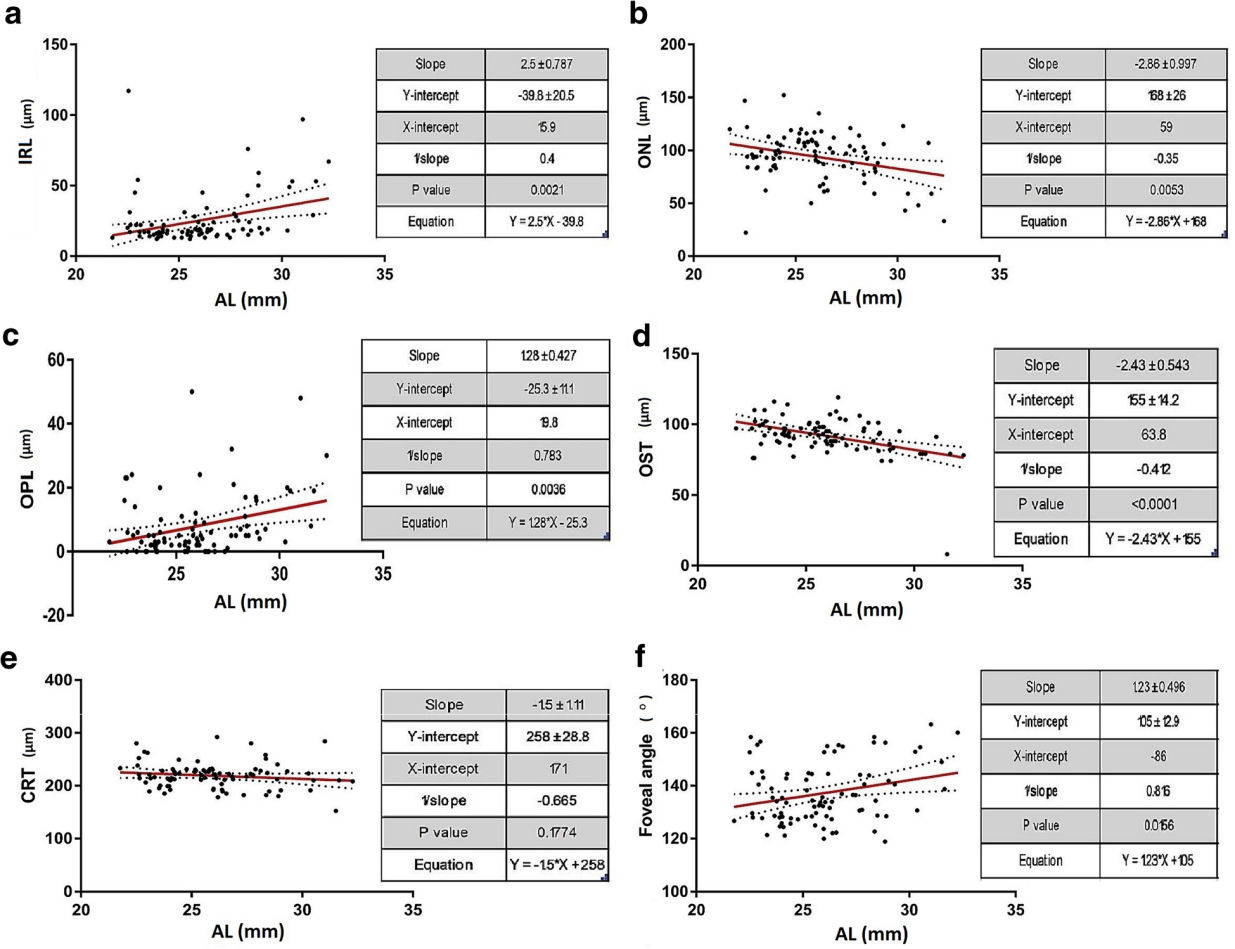
Correlation between retinal structure and axial length. **a**-**f** Multivariable linear regression analysis between macular retinal layer thicknesses (in μm) [inner retinal layer (IRL), outer nuclear layer (ONL), outer plexiform layer (OPL), outer segment thickness (OST), central retinal thickness (CRT)] and foveal angle (in °) and axial length (AL) (in mm). Linear regressions are shown with 95% confidence intervals for slopes

**Fig. 5:**
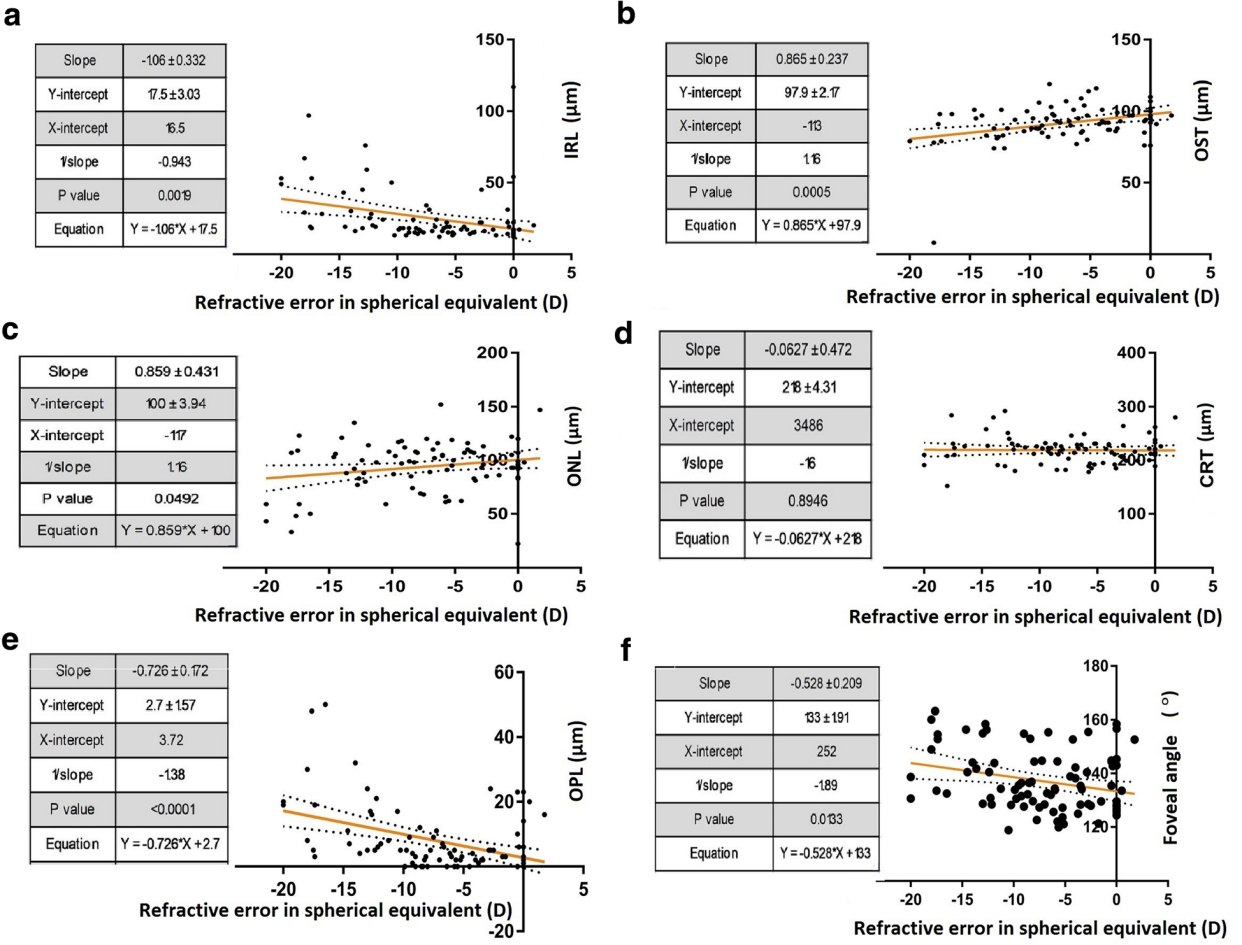
Correlation between retinal structure and refractive error. **a**-**f** Multivariable linear regression analysis between macular retinal layer thicknesses (in μm) [inner retinal layer (IRL), outer nuclear layer (ONL), outer plexiform layer (OPL), outer segment thickness (OST), central retinal thickness (CRT)] and foveal angle (in °) and spherical equivalent (SE) (in D). Linear regressions are shown with 95% confidence intervals for slopes

**Fig. 6:**
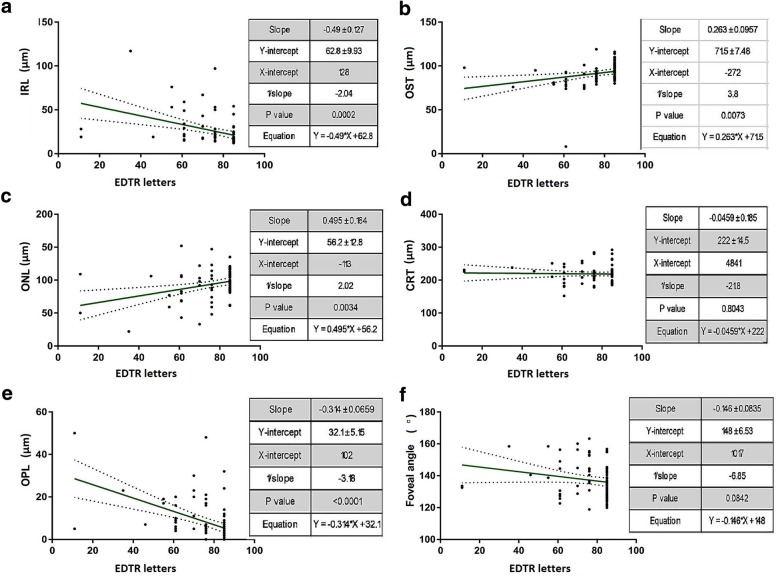
Correlation between retinal structure and visual function. **a**-**f** Multivariable linear regression analysis between macular retinal layer thicknesses (in μm) [inner retinal layer (IRL), outer nuclear layer (ONL), outer plexiform layer (OPL), outer segment thickness (OST), central retinal thickness (CRT)] and foveal angle (in °) and visual acuity (VA) (in ETDRS letters). Linear regressions are shown with 95% confidence intervals for slopes

For the study, individual retinal layer thicknesses at the macular region were measured using the automated layer segmentation algorithm in Spectralis, Heidelberg spectral domain OCT. The correlations between different macular layer thicknesses and AL, SE and VA are summarised in Table 2 and Figs. 4, 5 and 6. Analysis of structures with AL showed significant positive correlations with NFL (r = 0.828, *p* < 0.001), GCL (r = 0.772, *p* = 0.001), IPL (r = 0.699, *p* = 0.004), INL (r = 0.755, *p* = 0.001), IRL (r = 0.764, p = 0.001) and OPL (r = 0.771, *p* = 0.001) and significant negative correlations with ONL (r = − 0.560, *p* = 0.030) and photoreceptor OS thickness (r = − 0.856, *p* < 0.001). However, there was no correlation between AL and total retinal thickness at the macula (r = 0.388, *p* = 0.153). Significant positive correlations were observed between spherical equivalents and OS (r = 0.809, *p* < 0.001) while negative correlations were noted with NFL (r = − 0.747, *p* = 0.001), GCL (r = − 0.649, *p* = 0.009), IPL (r = − 0.631, *p* = 0.012) INL (r = − 0.680, *p* = 0.005), IRL (r = − 0.668, *p* = 0.007) and OPL (r = − 0.707, *p* = 0.003). Table 2 also shows the correlations between visual acuity and individual retinal layer thicknesses. Higher number of ETDRS letters were associated with thinner NFL (r = − 0.895, *p* < 0.001), GCL (r = − 0.898, *p* < 0.001), IPL (r = − 0.860, *p* < 0.001) INL (r = − 0.919, *p* < 0.001), IRL (r = − 0.909, *p* < 0.001) and OPL (r = − 0.899, p < 0.001) and thicker ONL (r = 0.615, *p* = 0.015) and OS (r = 0.733, *p* = 0.002).

**Table 2:** Correlations of macular retinal layers and foveal angle with axial length, spherical equivalent and visual acuity

	AL (mm)		SE (D)		VA (letters)	
	r value	*p* value	r value	*p* value	r value	*p* value
NFL (μm)	0.828	< 0.001	−0.747	0.001	−0.895	< 0.001
GCL (μm)	0.772	0.001	−0.649	0.009	−0.898	< 0.001
IPL (μm)	0.699	0.004	−0.631	0.012	−0.86	< 0.001
INL (μm)	0.755	0.001	−0.68	0.005	−0.919	< 0.001
IRL (μm)	0.764	0.001	−0.668	0.007	−0.909	< 0.001
OPL (μm)	0.771	0.001	−0.707	0.003	−0.899	< 0.001
ONL (μm)	−0.56	0.03	0.433	0.107	0.615	0.015
OS (μm)	−0.856	< 0.001	0.809	< 0.001	0.733	0.002
CRT (μm)	0.388	0.153	−0.186	0.508	−0.372	0.173
FA (°)	0.757	0.001	−0.635	0.011	−0.827	< 0.001

*AL* Axial length, *SE* Spherical equivalent, *VA* Visual acuity, *NF*L Nerve fibre layer, *GCL* ganglion cell layer, *IPL* Inner plexiform layer, *INL* Inner nuclear layer, *IRL* Inner retinal layer, *OPL* outer plexiform layer, *ONL* Outer nuclear layer, *OS* outer segment layer, *CRT* central retinal thickness, *FA*  Foveal angle

### Relationship between foveal angle and AL, SE and VA

**Fig. 7:**
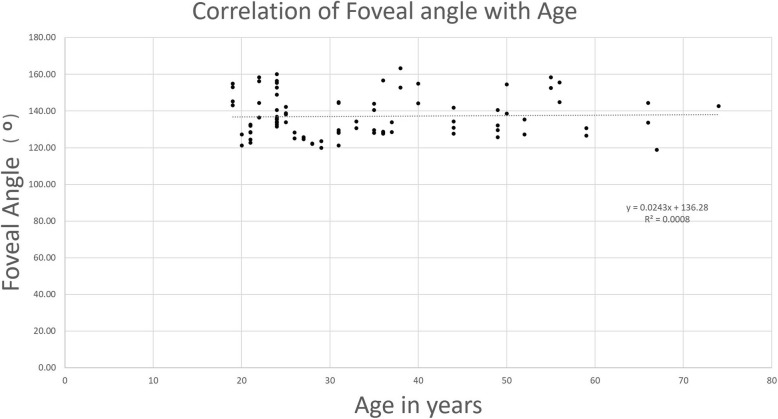
Correlation between foveal anatomy and age. Linear regression analysis between foveal angle (in °) and age (in years)

No correlation was observed between foveal angle measurements with age (Fig. 7). Foveal angle was more obtuse with increasing axial length (r = 0.757, *p* = 0.001) while reduced foveal angle was associated with lower spherical equivalents (r = − 0.635, *p* = 0.011) and better visual acuities (r = − 0.827, *p* < 0.001) (Table 2 and Figs. 4, 5 and 6).

### Relationship between OCTA findings and AL, SE and VA

**Fig. 8:**
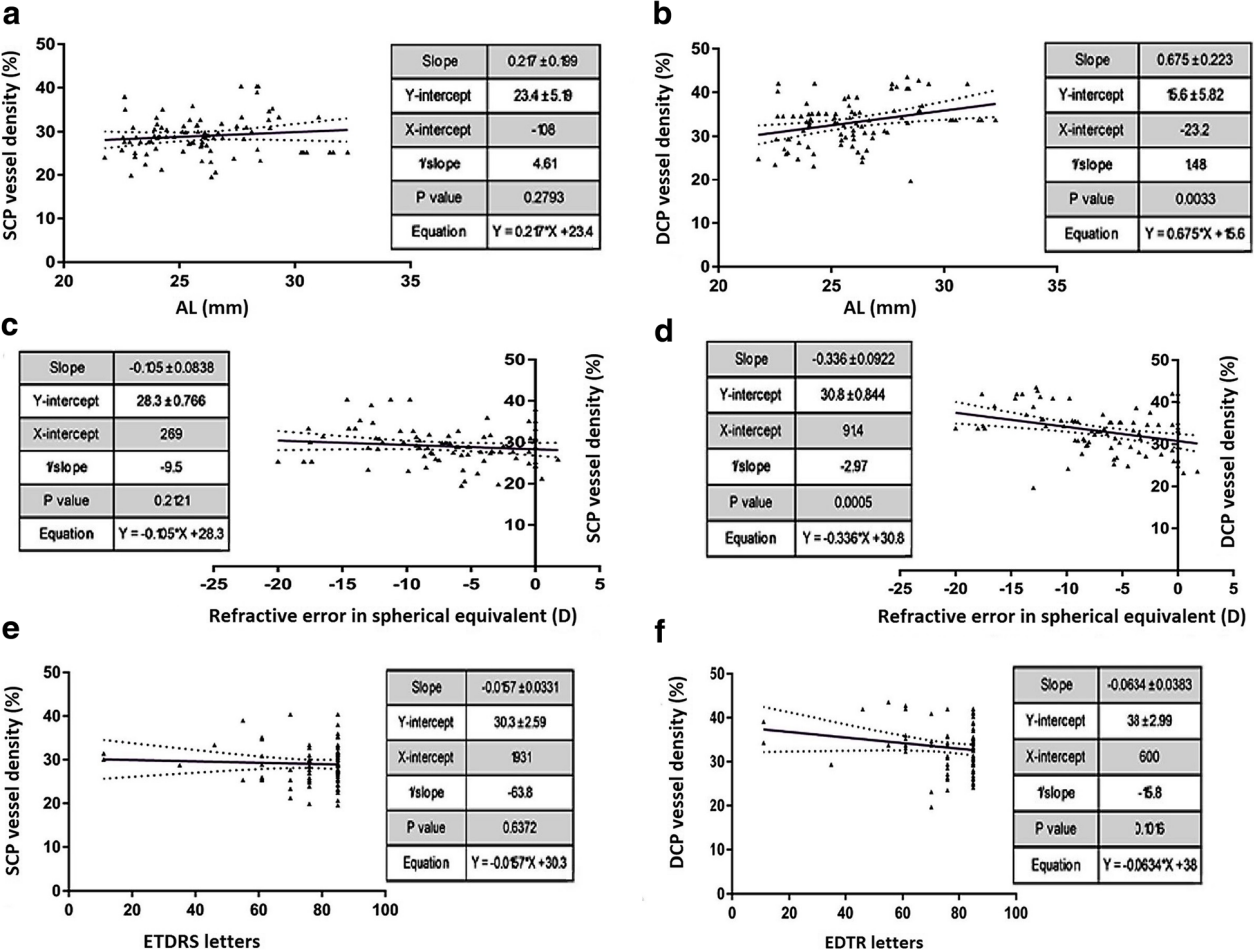
Correlation between retinal vasculature and axial length, refractive error and visual function. Multivariable linear regression analysis between vessel densities (in %) in superficial capillary plexus (SCP) and deep capillary plexus (DCP) and axial length (AL) (**a**,**b**), spherical equivalent (SE) (**c**,**d**) and visual acuity (VA) (**e**,**f**). Linear regressions are shown with 95% confidence intervals for slopes

Retinal vessel imaging was performed using the Avanti spectral domain OCT (RTVue-XR Avanti; Optovue, Fremont, CA, USA) and measurements were calculated with a built-in AngioVue software. Significant correlation was observed between vessel densities at the deep capillary plexus with axial length and spherical equivalent. The flow areas in the SCP and DCP and vessel densities in the SCP did not show statistically significant correlations with either AL, SE or VA (Table 3 and Fig. 8).

**Table 3:** Correlations between optical coherence tomography angiography indices with axial length, spherical equivalent and visual acuity using the Pearson’s correlation test

	Axial length (mm)		Spherical Equivalent (D)		Visual acuity (letters)	
	r value	*p* value	*r* value	*p* value	*r* value	*p* value
Flow index SCP	0.187	0.085	− 0.146	0.181	0.005	0.964
SCP Vessel density (%)	0.118	0.279	−0.136	0.212	−0.052	0.637
Flow index DCP	0.142	0.191	−0.093	0.395	−0.041	0.711
DCP Vessel density (%)	0.313	0.003	−0.37	< 0.001	−0.178	0.102

*SCP* Superficial capillary plexus, *DCP* Deep capillary plexus

## Discussion

Our study supported the hypothesis that with flattening of the foveal contour, inner retinal layer thickening, outer retinal layer thinning and changes in the deep vascular plexus, there is reduction in visual acuity with higher grades of myopia. In the present study, the retinal thicknesses at the macula were increased in NFL, GCL, IPL, INL and OPL while thinning of the ONL and OS layers were noted with increasing AL. The results of our study contrasted with that reported by Liu et al. [39] According to the authors, the central outer retinal layers, namely the myoid-ellipsoid zone (MEZ) layer and OS layer were found to be thickened with increasing AL. They speculated that the choroidal dysfunction with higher grades of myopia to be responsible for the outer retinal layer thickening. This choroidal dysfunction may affect the function of the retinal pigment epithelium thus leading to cell apoptosis of the OS and MEZ photoreceptor layers. The disturbed renewal of the photoreceptor OS/MEZ may result in OS elongation, apoptosis of cone cells, and subsequent thinning of the ONL [40]. We speculate that the retinal changes seen in our study may result from the combined tangential and/or antero-posterior tractional forces acting on the inner retina and tangential stretching force on the outer retina due to the overall myopic globe expansion. A similar mechanism has been described in eyes with myopic tractional maculopathy [41]. However, further longitudinal studies would be required to see whether these eyes progress to develop myopic tractional maculopathy. In the present study, we found no correlation between the total central retinal thickness and myopia (r = 0.388, *p* = 0.153), which is consistent with the results reported in previous studies [15, 42].

We analysed the foveal contour by measuring the foveal angle in our study. We found that with increasing AL, there was flattening of the foveal contour making the foveal angle more obtuse. This is explained by the same tractional and tangential forces acting on the inner retina. Similar findings of shallowing of foveal contour with persistent/thickening of IRLs have also been noted in ocular pathologies like retinopathy of prematurity and familial exudative vitreo-retinopathy [41–44]. These diseases are associated with secondary high myopia and the retinal findings could be because of high myopia rather than the primary disease itself.

Significant changes in retinal microvasculature in myopic eyes have demonstrated its effects on VA. There was a statistically significant positive correlation between the outer nuclear and outer segment layer thicknesses and VA in higher grades of myopia. This explains that photoreceptors which are primarily responsible for the visual function derive their blood supply from the choroidal circulation. In myopia, there is choroidal dysfunction leading to reduction in choroidal blood flow as identified by an increase in flow void areas seen on OCTA [34, 40]. As a result, there is reduction in blood supply to the photoreceptors causing thinning of the outer segment and outer nuclear layers and subsequent reduction in visual acuity. As we had included very high myopes in our study, the visual acuity may be affected by amblyopia as well. In such a case, the correlations of visual acuity could have been fallacious.

Various studies performing retinal vessel imaging on OCTA and analysing the flow void areas and vessel densities in myopic eyes have been published in literature [28–34]. There is a documented reduction in retinal micro vessel density in macular and peripapillary areas in myopic eyes compared to emmetropic eyes [28–32]. However, in our study, we found a positive correlation between the vessel density and flow area index in both the SCP and DCP with increasing AL and myopic refraction. This contrasts with that described by the previous studies. A possible explanation for this finding is as follows: The current OCTA nomenclature shows the SCP to be anatomically located within the NFL, GCL and IPL and the DCP within the INL and OPL [45]. In our study, we found persistence and thickening of the IRLs and OPLs in eyes with longer AL and high myopic spherical refraction. As a result, there is persistence or increase in both the SCP and DCP vasculature. Thus, higher vessel densities and flow area indices are noted in both SCP and DCP with higher grades of myopia. Falavarjani et al. reported increased vessel densities and reduction of FAZ area on OCTA in eyes of children born preterm compared to controls [43]. They speculated that the increase in the inner retinal layer is responsible for the increase in vessel densities on OCTA in these eyes. It was also noted that there was no correlation between the vessel densities on OCTA in SCP (r = − 0.052, *p* = 0.637) and DCP (r = − 0.178, *p* = 0.102) and visual acuity. Al-Sheikh et al. found significant reduction in vessel density and increase in flow void areas in the choriocapillaris layer of greater myopic eyes [34]. Thus, the reduction in visual acuity in myopic eyes is mainly due to the reduction in choroidal circulation with almost no contribution from the retinal circulation.

The major advantage of our study was the simultaneous correlation of retinal microstructure, microvasculature and visual function in myopic eyes. Furthermore, we analysed the foveal contour with different grades of myopia. Yet, our study had a few limitations as well. The most important being the OCT scanning protocol used in the study. We evaluated only the thickness changes along the horizontal scans. Retinal pathology related to myopia may occur in other regions around the macula. This scan protocol may limit our understanding of these changes. Also, we did not measure the subfoveal choroidal thickness in our study. Our study was limited by its transversal design, the age range of the subjects, and sample number that was relatively small and also by the effects of manual segmentation and high refractive errors on the measurements. Other longitudinal studies with a greater age spectrum, larger sample, and the use of a 3-dimensional volume scan protocol could be more informative with respect to the retinal structure and blood flow in myopic eyes. In addition, our quantitative vascular density was not confirmed by another evaluation method, nor by other manufacturer’s instruments, although there is no gold standard for vascular density measurements that we can compare with our quantitative data. If new exploration procedures are developed, vascular changes in pathologically myopic eyes are still unclear.

## Conclusion

We found that flattening of the foveal contour, inner retinal layer thickening, outer retinal layer thinning and changes in the deep vascular plexus were associated with reduction in visual acuity in eyes with higher grades of myopia. Ultra-high-resolution OCT along with OCTA imaging of the retinal microvasculature is a simple, non-invasive, and practical technique for informative evaluation and understanding of the different underlying mechanisms of pathologic changes related to myopia, such as lacquer cracks, atrophy, myopic choroidal neovascularisation, tractional maculopathy and macular holes.

## Abbreviations

AL: Axial length
DCP: Deep capillary plexus
ETDRS: Early treatment diabetic retinopathy study
FAZ: Foveal avascular zone
GCL: Ganglion cell layer
ILM: Inner limiting membrane
INL: Inner nuclear layer
IOP: Intraocular pressure
IPL: Inner plexiform layer
NFL: Nerve fibre layer
OCT: Optical coherence tomography
OCTA: Optical coherence tomography angiography
ONL: Outer nuclear layer
OPL: Outer plexiform layer
OS: Outer segment
SCP: Superficial capillary plexus
SE: Spherical equivalent
VA: Visual acuity
